# Prevalence of Plasmid-Mediated Quinolone Resistance Determinants and OqxAB Efflux Pumps among Extended-Spectrum **β**-Lactamase Producing *Klebsiella pneumoniae* Isolated from Patients with Nosocomial Urinary Tract Infection in Tehran, Iran

**DOI:** 10.1155/2015/518167

**Published:** 2015-08-02

**Authors:** Mehdi Goudarzi, Mehdi Azad, Sima Sadat Seyedjavadi

**Affiliations:** ^1^Infectious Diseases and Tropical Medicine Research Center, Shahid Beheshti University of Medical Sciences, Tehran 1985717443, Iran; ^2^Department of Microbiology, School of Medicine, Shahid Beheshti University of Medical Sciences, Tehran 1985717443, Iran; ^3^Department of Medical Laboratory Sciences, Faculty of Allied Medicine, Qazvin University of Medical Sciences, Qazvin 1651135779, Iran; ^4^Department of Medical Mycology, Pasteur Institute of Iran, Tehran 1316943551, Iran

## Abstract

*Objective*. Plasmid-mediated quinolone resistance (PMQR) plays an important role in the development of clinical resistance to quinolone. The aim of this study was to investigate PMQR determinants among extended-spectrum *β*-lactamases- (ESBL-) producing *Klebsiella pneumoniae* recovered from patients with nosocomial urinary tract infection (UTI). *Methods*. A total of 247 ESBL-producing *K. pneumoniae* isolates were collected from 750 patients with UTI. ESBL production was confirmed by double disc synergy test and combined disc diffusion test. The prevalence of PMQR determinants among ESBL-producing *K. pneumoniae* was assessed using PCR method. *Results*. The rates of resistance to antimicrobial agents in present study varied from 14.2% to 98.8%. In comparison with other PMQR genotypes, the frequency of *aac(6′)-Ib* (68.8%) was strikingly high. Of the 247 isolates tested, *qnrA, qnrB, qnrS,* and *qepA* genes were present in 3.6%, 1.6%, 1.2, and 2%, respectively. *oqxA* and *oqxB* were detected in 56.7% and 54.6% of isolates. The predominant coexisting ESBL and PMQR profile among our isolates included *bla*_CTX-M_ and *aac(6′)-Ib, oqxA, oqxB* (28.3%) and *bla*_TEM_, *bla*_SHV_ and *aac(6′)-Ib, oqxA,* and *oqxB* (19.4%) profile.  *Conclusion*. Given the linkage observed between resistance to quinolones and beta lactam antibiotics, therapeutic protocol with fluoroquinolones and beta lactam antibiotics should be seriously revised in Tehran hospitals.

## 1. Introduction

*K. pneumoniae* is common nosocomial pathogen causing urinary tract infection in different wards of hospital including infectious, surgical, and intensive care unit. During the last decade transferable multidrug resistance in Gram-negative bacteria, particularly* K. pneumoniae* isolates, has become an escalating global threat [1]. Beta lactam resistance is mediated by acquisition of *β*-lactamase genes that are mostly plasmid encoded. Based on several investigators idea, plasmid-encoded temoneira (TEM), sulfhydryl variable (SHV), and cefotaximase (CTX-M) are the most prevalent ESBLs [2, 3].

Quinolone resistance among* K. pneumoniae* clinical isolates became a serious problem in developing countries as well as in developed countries, since the quinolones as broad-spectrum antimicrobial agents are widely prescribed for treatment of UTI caused by ESBL-producing* K. pneumoniae*. For years, it was assumed that quinolone resistance among Enterobacteriaceae is only originated through chromosomal mutations in the quinolone resistance-determining regions (QRDRs) including DNA gyrase, DNA topoisomerase IV, and genes coding for outer membrane proteins but plasmid-mediated quinolone resistance (PMQR) has been discovered recently. PMQR confers low-level resistance to quinolone [4]. Plasmid-mediated quinolone resistance (PMQR) was reported first from a* K. pneumoniae* isolate from the United States in 1998 [5].

Three mechanisms of PMQR including* qnr*,* aac(6*′*)-Ib-cr*, and active efflux pumps have been discovered in clinical isolates [4]. Qnr family (*QnrA*,* QnrB*,* QnrS*,* QnrC*, and* QnrD*) leads to quinolone resistance by protecting DNA gyrase and topoisomerase IV [5]. The* aac(6*′*)-Ib-cr* gene by adding an acetyl group to aminoglycoside modifies not only aminoglycosides but also fluoroquinolones. QepA is a quinolone efflux pump protein and leads to decrease of susceptibility to quinolones especially norfloxacin, ciprofloxacin, and enrofloxacin.* OqxAB*, a multidrug efflux pump, is related to reduced fluoroquinolone susceptibility and resistance to multiple agents [6–9]. The existence of multiple resistance genes on the same plasmid and their transfer between ESBL-producing clinical isolates has increased markedly, so that some reports demonstrated a strong relationship of quinolone resistance with the production of ESBLs and AmpC beta-lactamase [10]. Researches on the prevalence of PMQR genes and their different types in ESBL-producing* K. pneumoniae* isolated from patient with nosocomial UTI in our country are very sparse. This study could provide information about understanding prevalence and also dissemination PMQR determinants among ESBL-producing* K. pneumoniae* recovered from patients with nosocomial UTI in Iran.

## 2. Materials and Methods

### 2.1. Study Setting and Bacterial Isolates

This investigation was conducted at the Shahid Beheshti Unversity of Medical Sciences. Samples were collected from general hospitals in Tehran. In this descriptive study, 310 (41.3%)* K. pneumoniae* clinical isolates were collected from 750 urine specimens of hospitalized patients with UTI from wards of different hospitals in Tehran during the period of 10 months from July 2014 to April 2015. Of 310* K. pneumoniae* clinical isolates, 247 isolates (79.7%) were ESBL producers and were included in our study. Nosocomial UTI was confirmed through clinical examination conducted by a physician to exclude community-acquired infections. All the urine specimens were immediately transported to the laboratory and identified by routine conventional biochemical and microbiological tests. Colony count semiquantitative method was performed according to surface streak procedure using calibrated loops. Incubation was done in aerobic conditions at 37°C for 24–48 hours. The result of equal to or more than 10^5^ CFU/mL was considered as positive UTI [15]. Confirmed samples were maintained in Tryptic Soy Broth (TSB; Merck, Germany) containing 15% glycerol at −70°C until use.

### 2.2. ESBL Confirmatory Test

According to the Clinical and Laboratory Standards Institute (CLSI) criteria for ESBL screening, double disc synergy test (DDST) and combined disk diffusion test (CDDT) were performed [16]. All the ESBL isolates were also screened for* bla* genes (SHV, TEM, and CTX-M) using PCR.

#### 2.2.1. Double Disc Synergy Test (DDST)

DDST was done as a standard disk diffusion assay by using cefotaxime (30 *μ*g) and ceftazidime (30 *μ*g) with and without clavulanic acid (10 *μ*g) discs on Mueller-Hinton agar (MHA, Oxoid, United Kingdom) with 25 mm apart from each other. The organism was regarded as positive for the ESBL production when the zone of inhibition was equal to or more than 5 mm for either antimicrobial agent tested with clavulanic acid or its zone when tested without clavulanic acid [17].

#### 2.2.2. Combined Disc Diffusion Test (CDDT)

CDDT was done by using both ceftazidime (30 *μ*g) discs alone and in combination with clavulanic acid (30 *μ*g/10 *μ*g). Discs were placed 25 mm apart from each other. The inhibition zone around the ceftazidime disc combined with clavulanic acid was compared with the zone around the disc with the ceftazidime alone. Enhancement of the inhibition zone diameter being more than or equal to 5 mm for either antimicrobial agent tested in combination with clavulanic acid or its zone when tested alone, indicating synergy between clavulanic acid and antimicrobial agent tested, was regarded as ESBL positive [18].* E. coli* ATCC 25922 and* K. pneumoniae* ATCC 700603 were used as quality control.

### 2.3. Antimicrobial Susceptibility Testing

To evaluate susceptibilities of ESBL-producing* K. pneumoniae* isolates to 15 antimicrobial agents, routine disc diffusion susceptibility testing by Kirby-Bauer Disk diffusion method was performed according to CLSI recommendations [17]. Antimicrobial discs supplied by HiMedia Laboratories Pvt., Ltd., Mumbai, India, were including ceftriaxone (CTR 30 *μ*g), cefpodoxime (CPD 10 *μ*g), tetracycline (TE 30 *μ*g), tigecycline (TGC 15 *μ*g), aztreonam (AT 30 *μ*g), gentamicin (GEN 10 *μ*g), ciprofloxacin (CIP 5 *μ*g), amikacin (AK 30 *μ*g), ceftazidime (CZX 30 *μ*g), imipenem (IMP 10 *μ*g), cefotaxime (CTX 30 *μ*g), piperacillin (PI 100 *μ*g), cotrimoxazole (COT 25 *μ*g), norfloxacin (NX 10 *μ*g), and nalidixic acid (NA 30 *μ*g).* E. coli* ATCC 25922 was used as quality control strain in susceptibility testing. The confirmed samples as* K. pneumoniae* were stored at −70°C in Tryptic Soy Broth (TSB; Merck, Germany) containing 20% glycerol and were subjected to molecular identification.

### 2.4. DNA Extraction and PCR Assay

DNA extraction was performed using the QIAamp DNA isolation kit (Qiagen, Hilden, Germany) precisely according to the manufacturer's recommendation. After DNA extraction, the concentration of DNA was assessed by spectrophotometer. Detection of ESBL and PMQR genes was carried out using PCR amplification with degenerate primers listed in Table 1. The amplicons were separated on 1.2% agarose gel (Invitrogen, Carlsbad, CA, USA) prepared in TAE buffer at 80 V for 2 h and then visualized using ultraviolet light (UVItec, Cambridge, UK) after staining with Ethidium Bromide. Both strands of the purified amplicons were sequenced and compared with genes in the GenBank using the BLAST program (http://www.ncbi.nlm.nih.gov/BLAST/) to confirm their identities.

### 2.5. Statistical Analysis

In this research, specificity of the primer pairs was evaluated by using Gene Runner software (Hastings Software, Hastings-on-Hudson, NY, USA). SPSS software for Windows, version 17.0 (SPSS Inc., Chicago, IL), was used for statistical analysis.

## 3. Results

### 3.1. Prevalence of ESBL-Producing* K. pneumoniae* Isolates

In this study 310* K. pneumoniae* clinical isolates were collected from 750 urine specimens of hospitalized patients with UTI. Of 310* K. pneumoniae* clinical isolates 247 isolates (79.7%) were ESBL producers. Of the 247 isolates included in the study, 208 isolates (84.2%) had been isolated from males and 39 isolates (15.8%) had been isolated from females (M : F ratio was 0.18). The range of patient's age varied from 4 months to 65 years old with a median of 42.8 years. The patient age distribution in our study was 9.7% being equal to or less than 15 years, 71.7% from 16 to 35 years, 7.7% from 36 to 50 years, and 10.9% being equal to or greater than 60 years. The prevalence of ESBL-producing* K. pneumoniae *isolates in 750 urine specimens obtained from hospitalized patients with UTI was estimated to be 32.9% (247 isolates) by DDST and CDDT phenotypic methods.

### 3.2. Antimicrobial Drug Susceptibility

The result of the antimicrobial susceptibility testing of 247* K. pneumoniae* clinical isolates to 15 antibiotics tested revealed high frequency of the resistance to the majority of antimicrobial agents tested. As the results of this study showed, the resistance rates of isolates to tested antibiotics were ceftazidime 81.4%; norfloxacin 80.5% and cefotaxime 79.7%; cefpodoxime 76.2% and cotrimoxazole 72.1%; aztreonam 68% and ceftriaxone 64.8%; piperacillin 64.8%; nalidixic acid 62.8%; ciprofloxacin 60.8%; gentamicin 57.9%; tetracycline 48.6%. The highest susceptibility rate was found for tigecycline (85.8%), amikacin (62.8%), and imipenem (60.7%), respectively. In vitro, the susceptibilities of ESBL-producing strains to 17 antibiotics are shown in Table 2. Multidrug-resistance (MDR) was defined as resistance to at least three or more unrelated antibiotics [19]. Of 247 isolates, 215 (87%) were MDR. Surprisingly, out of 215 MDR ESBL-producing* K. pneumoniae*, 63 isolates (29.3%) were simultaneously resistant to cefpodoxime, norfloxacin, nalidixic acid, and cotrimoxazole. The predominant resistance profile among our isolates was resistance to 7 antibiotics (46.5%).

### 3.3. Prevalence of* bla* and PMQR Genes

The PCR results of* bla*  gene revealed a dominant presence of *bla*_CTX-M_ (74.9%) in our study that was followed by *bla*_TEM_ (70%) and *bla*_SHV_ (59.9%), respectively. The presence of plasmid-mediated quinolone resistance genes was evaluated among 247 ESBL positive* K. pneumoniae* isolates. The existence of* aac(6*′*)-Ib* was confirmed by sequencing in 148 isolates (68.8%),* oqxA* in 140 isolates (56.7%),* oqxB* in 130 isolates (54.6%),* qnrA* in 9 isolates (3.6%),* qnrB* in 4 isolates (1.6%),and* qnrS* in 3 isolates (1.2%). Coexistence of ESBL and PMQR genes was identified in 172 (69.6%)* K. pneumoniae* isolates. Coexistence of *bla*_CTX-M_ and* aac(6*′*)-Ib*,* oqxA, *and* oqxB* was the most widely distributed resistance genotype, which was observed in 70 (40.7%) isolates. The distribution of ESBL-encoding genes and PMQR genes and also their coexistence among 247 ESBL producers is shown in Tables 3 and 4.

## 4. Discussion

Beta lactam and quinolone antibiotics are the most important antimicrobial agents used for the treatment of* K. pneumoniae* infections. The inappropriate use of these antibiotics recently has led to the spread of MDR among* K. pneumoniae* isolates. The increase of resistance isolates to beta lactam and quinolone antibiotics is an escalating global threat for the empirical treatment of nosocomial infections [20]. Although chromosomal QRDR mutations in topoisomerases play an important role in conferring a high level of quinolone resistance, some of researchers believed that PMQR may contribute to an increase in quinolone resistance in clinical isolates of Enterobacteriaceae [5]. In recent years, increase of ESBL-producing* K. pneumoniae* isolates has been reported worldwide. High prevalence of ESBL-producing* K. pneumoniae* has been reported by various investigators. In a study that was done by Feizabadi et al. in 2010, 89* K. pneumoniae* isolated from hospitalized patients were investigated. They exhibited that 62 isolates (69.7%) were ESBL producers and all isolates were susceptible to imipenem. Aztronam and amoxiclave (with the resistance rate of 79.7%) were the least effective antibiotics against isolates of* K. pneumoniae* in their study. They found that *bla*_SHV_ (*n* = 60, 67.4%) was the most prevalent gene detected and followed by *bla*_TEM_ (*n* = 48, 54%), *bla*_CTX-M-I_ (*n* = 34, 38.2%), and *bla*_CTX-M-III_ (*n* = 24, 27%) [3]. Although overall trend of ESBL* K. pneumoniae* is on the rise, there are considerable geographical differences in the prevalence of ESBL-producing* K. pneumoniae* and also their ESBL genotypes. Our results showed a very high prevalence of ESBL-producing* K. pneumoniae* (79.7%) while in Latin America 45% [21], Philadelphia 75.8% [22], Venezuela 47.6% [23], Spain 20.8% [24], Nigeria 38% [25], South Korea 16.8% [6], Turkey 78.6% [26], Saudi Arabia 55% [27], India 17% [28] and China 51% [29] of* K. pneumoniae* isolates were reported to be ESBL producers. According to the large survey done in 10 European countries, the prevalence of ESBL producers ranged from as low as 1.5% in Germany to as high as 39–47% in Russia, Poland, and Turkey [30]. These differences depend upon various factors like type of samples, design of study, and carriage rate among the hospital personal and antibiotic policy.

In our study all isolates were resistant to penicillin and the majority of our isolates were resistant to other beta lactam antibiotics as well as quinolone. These findings are consistent with other studies [20, 31]. Although predominant genotypes of ESBLs in different studies are diverse, as expected, in present study CTX-M enzymes were the most prevalent type of ESBL (74.9%) followed by TEM (70%) and SHV (59.9%). Our results were in accordance with the study conducted in Lithuania, by Šeputiene et al., that showed that the prevalence of CTX-M in comparison with other ESBLs genotypes among* K. pneumoniae* isolates was relatively high (71%) [32]. Newly, some studies exhibited that the predominant genotypes of ESBLs in different areas were diverse; for instance, in Italy, Portugal, and Turkey, the most predominant ESBL genotype was TEM. Resistance to quinolones has created serious therapeutic problems. Resistance to quinolone in ESBL-producing isolates has been previously reported [4, 20]. As previously mentioned PMQR genes play an important role in resistance to quinolone due to its horizontal transferability [33]. It should be noted that prevalence of PMQR genes among our isolates was high (89.1%) and represents a potential reservoir for the spread of these genes. These results are in accordance with study conducted by Pasom et al. in Thailand [34]. In our study, the most prevalent of PMQR gene was* aac(6*′*)-Ib* (68.8) followed by* oqxA* (56.7%),* oqxB* (54.6%),* qnr* (6.4%), and* qepA* (2%). High frequency of* aac(6*′*)-Ib* among ESBL producers has been reported previously. The study was conducted in 2011 in Spain; out of 382 isolates of ESBL-producing* K. pneumoniae* and* Escherichia coli* investigated, 14 isolates (3.7%) were positive for* qnr* genes and 62 isolates (16.2%) were positive for the mutant variant of* aac(6*′*)-Ib-cr*. They exhibited that* aac(6*′*)-Ib-cr* gene was the most prevalent PMQR gene in* E. coli* and* K. pneumoniae* producing ESBL [35]. However, high frequency of* aac(6*′*)-Ib* among our isolates supports earlier suggestions in which* aac(6*′*)-Ib-cr* genes are coassociated with genes encoding ESBLs [36].

The data from our investigation showed that the percentage of* qnr* genes among ESBL positive* K. pneumoniae* was 6.4%. In China 65.5% [37], Malaysia 48.9% [38], USA 11.1% [39], Singapore 5.2% [40], and Brazil 2.3% [41] of* K. pneumoniae* isolates were reported to be* qnr* positive strains.

*OqxAB*, as a multidrug efflux pump, confers resistance to quinolone such as ciprofloxacin, flumequine, norfloxacin, and nalidixic acid and other antibiotics such as chloramphenicol and trimethoprim in clinic [4]. The present study demonstrated prevalence of 56.7% and 54.6% for* oqxA* and* oqxB* genes among 274 ESBL-producing isolates of* K. pneumoniae* which was lower than those in Spain (76.3% for* oqxA* and 74.6% for* oqxB*) [9] and China (100% for both genes) [8]. The high rate of oqxAB gene might reflect inappropriate use of antimicrobial agents and even close contact with domestic animals could be presumptive cause for dissemination of* oqxAB* gene. In line with the results of other studies which found that* qepA* was rarely present in* K. pneumoniae* clinical isolates [42, 43], in our study,* qepA* was detected in 2% isolates.

## 5. Conclusion

In summary, the results of this study reflected considerable frequency of* aac(6*′*)-Ib* and efflux pump genes among ESBL positive* K. pneumoniae* clinical isolates in our hospitals. This high frequency of PMQR genes indicated that early detection and routine screening of ESBL-producing* K. pneumoniae* for the prevention of the development ESBL isolates PMQR carriage in our area are very important.

## Figures and Tables

**Table 1:** Primers sequence used to detect *bla* genes and PMQR genes in this study.

Target gene	Nucleotide sequence	Size of the amplified product (bp)	Reference
*bla*_TEM_	5′-TCGGGGAAATGTGCGCG-3′	972	[2]
	5′-TGCTTAATCAGTGAGGCACC-3′		

*bla*_SHV_	5′-GGGTTATTCTTATTTGTCGC-3′	615	[3]
	5′-TTAGCGTTGCCAGTGCTC-3′		

*bla*_CTX-M_	5′-ACGCTGTTGTTAGGAAGTG-3′	857	[3]
	5′-TTGAGGCTGGGTGAAGT-3′		

*aac(6*′*)-Ib *	5′-TTGCGATGCTCTATGAGTGGCTA-3′	482	[11]
	5′-CTCGAATGCCTGGCGTGTTT-3′		

*qepA *	5′-CTGCAGGTACTGCGTCATG-3′	403	[12]
	5′-CGTGTTGCTGGAGTTCTTC-3′		

*oqxA *	5′-GACAGCGTCGCACAGAATG-3′	339	[13]
	5′-GGAGACGAGGTTGGTATGGA-3′		

*oqxB *	5′-CGAAGAAAGACCTCCCTACCC-3′	240	[13]
	5′-CGCCGCCAATGAGATACA-3′		

*qnrA *	5′-AGAGGATTTCTCACGCCAGG-3′	619	[13]
	5′-GCAGCACTATKACTCCCAAGG-3′		

*qnrB *	5′-GATCGTGAAAGCCAGAAAGG-3′	469	[14]
	5′-CGATGCCTGGTAGTTGTCC-3′		

*qnrS *	5′-GCAAGTTCATTGAACAGGCT-3′	428	[12]
	5′-TCTAAACCGTCGAGTTCGGCG-3′		

**Table 2:** Antimicrobial susceptibly pattern of 247 ESBL producing *K. pneumonia*e isolated from patients with UTI to 15 antimicrobial agents.

Antibiotics	Antibiotic susceptibility (*n* = 247)		
	R	I	S
	*n* (%)	*n* (%)	*n* (%)
Cefotaxime	197 (79.7)	5 (2.1)	45 (18.2)
Ceftazidime	201 (81.4)	10 (4)	36 (14.6)
Ceftriaxone	160 (64.8)	4 (1.6)	83 (33.6)
Cefpodoxime	188 (76.2)	7 (2.8)	52 (21)
Piperacillin	160 (64.8)	0 (0)	87 (35.2)
Aztreonam	168 (68)	0 (0)	79 (32)
Imipenem	93 (37.7)	4 (1.6)	150 (60.7)
Amikacin	86 (34.8)	6 (2.4)	155 (62.8)
Gentamicin	143 (57.9)	15 (6.1)	89 (36)
Norfloxacin	199 (80.5)	4 (1.6)	44 (17.9)
Ciprofloxacin	150 (60.8)	6 (2.4)	91 (36.8)
Nalidixic acid	155 (62.8)	12 (4.8)	80 (32.4)
Cotrimoxazole	178 (72.1)	5 (2)	64 (25.9)
Tetracycline	120 (48.6)	8 (3.2)	119 (48.2)
Tigecycline	35 (14.2)	0 (0)	212 (85.8)

**Table 3:** Distribution of ESBL-encoding genes and PMQR genes among 247 ESBL producers.

Resistance genes	Number (%)
*bla*_CTX-M_	185 (74.9)
*bla*_TEM_	173 (70)
*aac(6*′*)-Ib *	170 (68.8)
*bla*_SHV_	148 (59.9)
*oqxA *	140 (56.7)
*oqxB *	130 (54.6)
*qnrA *	9 (3.6)
*qepA *	5 (2)
*qnrB *	4 (1.6)
*qnrS *	3 (1.2)

**Table 4:** Coexisting ESBL and PMQR resistance genes among 247 ESBL producers.

Coexisting resistance genes	Number (%)
*bla*_CTX-M_ and *bla*_TEM_	85 (34.4)
*bla*_CTX-M_ and *aac(6*′*)-Ib, oqxA,*and* oqxB *	70 (28.3)
*bla*_TEM_, *bla*_SHV_ and *aac(6*′*)-Ib*, *oqxA*, and *oqxB *	48 (19.4)
*bla*_CTX-M_ and *bla*_SHV_	24 (9.7)
*qnrA, oqxA,* and* oqxB *	7 (2.8)
*bla*_CTX-M_, *bla*_TEM_, *bla*_SHV_, and *aac(6*′*)-Ib *	4 (1.6)
*bla*_CTX-M_, *oqxA, oqxB,* and* qnrA *	2 (0.8)
*aac(6*′*)-Ib, oqxA, oqxB, qnrB, *and *qnrS *	1 (0.4)
